# A case report of hepatitis B virus reactivation 19 months after cessation of chemotherapy with rituximab

**DOI:** 10.3389/fimmu.2022.1083862

**Published:** 2022-12-02

**Authors:** Xiangjuan Guo, Tongtong Ji, Shengliang Xin, Jinghang Xu, Yanyan Yu

**Affiliations:** ^1^ Department of Infectious Diseases, Peking University First Hospital, Beijing, China; ^2^ Department of Infectious Diseases, Handan Central Hospital, Handan, China

**Keywords:** hepatitis B virus reactivation, lymphoma, rituximab, hepatitis B virus, CD20 monoclonal antibody

## Abstract

A 72-year-old woman presented to our hospital with elevation of serum transaminases. Her blood tests showed the hepatitis B surface antigen (HBsAg) and hepatitis B e antigen (HBeAg) negative. Rituximab plus cyclophosphamide, doxorubicin, vincristine, and prednisone (R-CHOP) were given for the diffuse large B-cell lymphoma. She didn’t receive anti- hepatitis B virus (HBV) drug for the isolated HBcAb positive. HBV reactivation confirmed based on the serum HBV DNA detectable until 19 months after stopping R-CHOP regimen. HBV DNA became undetectable after 4 weeks therapy with Tenofovir alafenamide fumarate (TAF). Serum transaminases went down to normal 3 months later after receiving TAF. HBV reactivation is a substantial risk for patients with isolated HBcAb positive receiving rituximab-containing chemotherapy without anti- HBV drug. Regular monitoring with a frequency of 1-3 months is the basis for timely diagnosis and treatment of HBV reactivation. Serum transaminases abnormalities may be the initial manifestation of HBV reactivation.

## Introduction

HBV reactivation is a well-known complication of HBV infected patients undergoing chemotherapy or immunosuppressive therapy. It is well documented (1–7) that prophylactic anti-HBV therapy should be given to HBsAg-negative, HBcAb-positive patients before receiving CD20 monoclonal antibody drugs, but there is no consensus on the duration of prophylactic medication, frequency of monitoring during and after prophylactic treatment, or monitoring indicators. The duration of prophylactic medication recommended by Chinese Society of Hepatology (5) and European Association for the Study of the Liver (EASL) (3) is at least 18 months since the cessation of immunosuppressive therapy. Here we report a case of HBV reactivation 19 months after cessation of chemotherapy with rituximab for diffuse large B-cell lymphoma in a patient with resolved hepatitis B infection (HBsAg negative, HBcAb positive and HBV DNA negative).

## Case report

72-year-old women presented to our hospital with elevation of serum transaminases for one day on August 19, 2021. Liver chemistry showed alanine aminotransferase (ALT) 132 U/L, aspartate aminotransferase (AST) 92 U/L, alkaline phosphatase(ALP) 113 U/L, γ-glutamyl transferase (GGT) 56 U/L. She has no fatigue, anorexia, nausea, abdominal pain, dark urine, pale stool, itchy skin, or other symptoms. Past medical history: She underwent splenectomy and was diagnosed with diffuse large B-cell lymphoma due to splenomegaly in September 2019. She started R-CHOP regimen in November 2019. Her laboratory finding showed isolated HBcAb positive (HBcAb+/HBsAb-/HBsAg-/HBeAb-/HBeAg-), undetected (< 20 IU/ml) HBV DNA, and normal transaminases before and during chemotherapy. Complete response to chemotherapy was achieved and immunosuppressive therapy was withdrawn in June 2020 after completing 4 cycles of it. Monitoring of HBV markers and aminotransferases every 1-3 months after chemotherapy showed no change through 14 months after cessation of chemotherapy. She had no history of receiving anti-HBV drug, liver diseases and blood transfusion. She has no history of taking Chinese traditional medicine, health products or other drugs. She had no history of alcohol consumption. Physical examination didn’t show any signs of liver disease (jaundice, liver palm, spider angioma, hepatomegaly, shifting dullness or edema in lower limbs). Blood tests still showed isolated HBcAb positive and undetected HBV DNA. The patient was tested negative for antibodies of hepatitis A virus (HAV) IgM, anti-hepatitis C virus (HCV) IgG, anti-hepatitis E virus (HEV) IgM and IgG. The serum Cytomegalovirus-DNA and Epstein Barr viruses-RNA were negative(<500copies/ml). Anti-nuclear antibody (ANA) was borderline positive (1:100); Anti-soluble liver antigen and anti-liver-pancreas antibodies (Anti-SLA/LP), anti-mitochondrial autoantibodies (AMA), liver kidney microsomal antigen (LKM) and smooth muscle antibody (SMA) were negative. Serum immunoglobulin G (IgG) and immunoglobulin M (IgM) were normal. Ultrasonography showed markedly increased echogenicity of the liver, thickening of the gallbladder wall, and right renal cyst. Fibro Scan showed an increased CAP score (300 dB/m) and a fibrosis score of 7.1 Kpa.

On August 19, 2021, she was prescribed diammonium glycyrrhizinate enteric-coated capsules and bicyclol tablets for liver protection. One month later (September-2021), ALT level decreased to normal (27 U/L). However, AST level kept abnormal. Four months later (January-2022), HBV DNA increased to 7.8 E+01 IU/ml. Although blood test on the same day still showed isolated HBcAb positive, HBV reactivation was considered, and antiviral therapy was recommended to her. Unfortunately, she refused anti-HBV therapy. She was advised to come back to the hospital for monthly follow-up, but she did not until April 2022.

**Figure 1:**
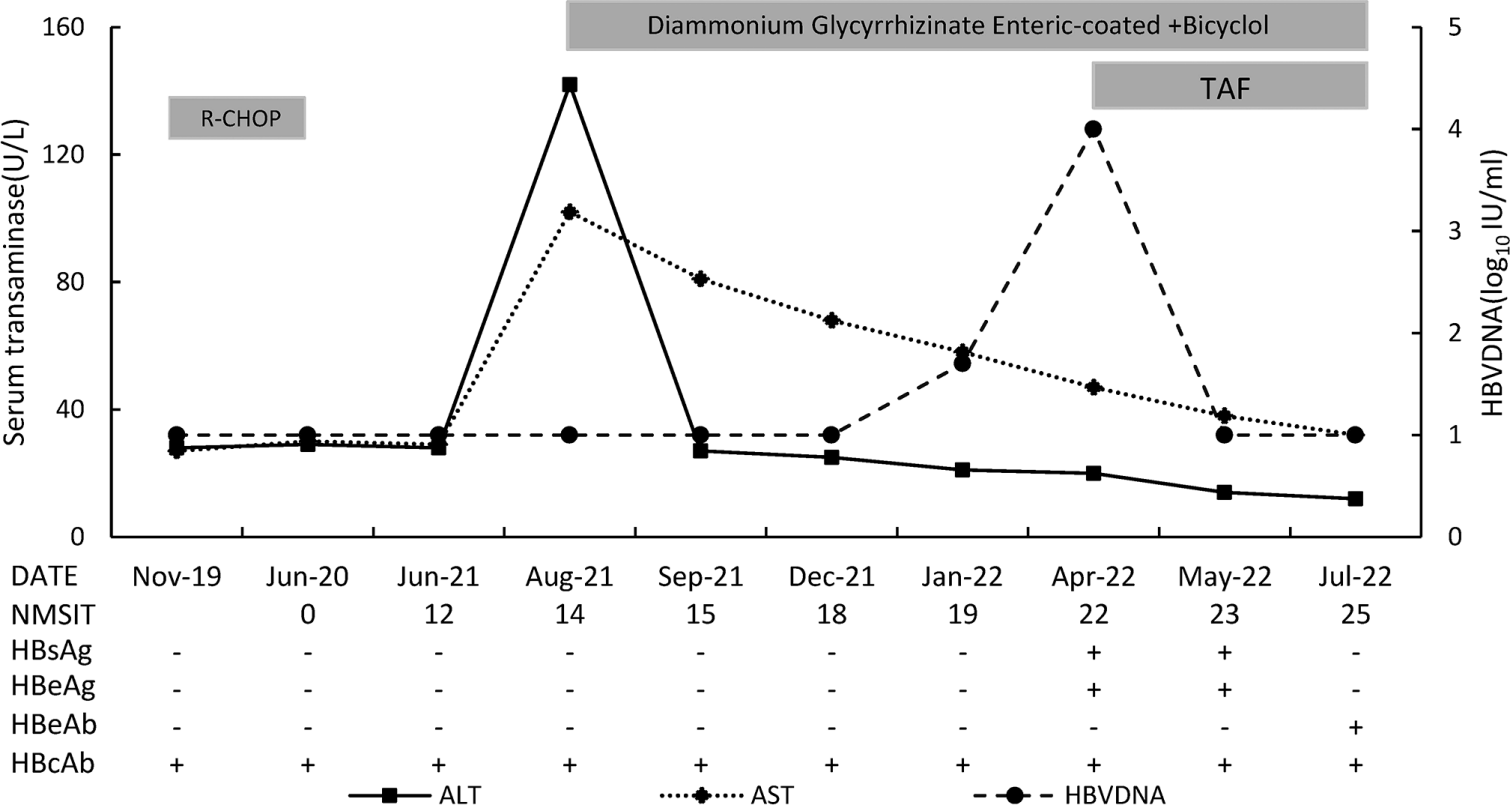
The sketch map of this patient. NMSIT: the number of months after stoping immunosuppressive therapy.

In April 2022, HBV DNA level increased significantly to 1.86 E+04 IU/ml, together with positive of HBsAg, HBeAg and HBcAb. Liver biochemistry showed ALT 20 U/L, AST 47 U/L. She was prescribed TAF immediately. One month later (May-2022), HBV DNA turn back to undetectable. Three months later (July-2022), complete response (undetectable HBV DNA, HBsAg loss and transaminase normalization) was achieved. Re-examined Fibro Scan in July 2022 showed a CAP score of 255 dB/m and a fibrosis score of 4.0 Kpa. The laboratory findings during the monitoring period are shown in **Figure 1**.

## Discussion

HBV reactivation is a substantial risk for patients with isolated HBcAb positive receiving rituximab-containing chemotherapy. Studies (8–12) showed that the incidence of HBV reactivation was 3%-68.3% in patients with isolated HBcAb positive who didn’t take prophylactic antiviral therapy while receiving rituximab. The incidence of liver failure after HBV reactivation ranges from 0% to 37.5%, resulting in a mortality rate of 0% to 12.5% (11, 13, 14). Prophylaxis anti-HBV therapy is recommended in current guidelines for lymphoma patients with isolated HBcAb positive who received R–CHOP. This patient did not receive anti-HBV drugs for prophylaxis prior to receiving rituximab and subsequently developed HBV reactivation and ALT flare. This case suggested a necessity of prophylactic antiviral therapy for the patients with isolated HBcAb positive in this clinical setting.

In addition, this case also reminds us that the time between stopping of immunosuppressive therapy and HBV reactivation can exceed 18 months, which is the course recommended by EASL and Chinese Society of Hepatology (3, 5) for antiviral prophylaxis in patients receiving B-cell monoclonal antibody therapy. Surprisingly, HBV reactivation happened 19 months after withdrawal of immunosuppressive therapy in this patient. Although HBV reactivation was reported (15) in a patient 55 months after treatment with autologous peripheral blood stem cell transplantation and R-CHOP chemotherapy, late HBV reactivation is rare in patients treated with R-CHOP regimen alone. Researches (16, 17) showed 83%-86% of HBV reactivation in individuals with isolated HBcAb positive receiving rituximab-containing chemotherapy occurred within 12 months after treatment with R-CHOP regimen. Therefore, both physicians and patients may ignore the necessity of subsequent monitoring for HBV reactivation 12 months after chemotherapy. The timely diagnosis of HBV reactivation becomes difficult when it appears 12 months or later after prophylactic therapy, resulting in a delay in intervention and even liver failure or death. So, this case reminds clinicians that long-term, at least 19 months, monitoring of HBV reactivation is required after discontinuation of rituximab therapy in individuals with isolated HBcAb positive.

Although the monitoring of HBV reactivation for patients with isolated HBcAb positive receiving rituximab-containing chemotherapy has become a focus of clinical attention, international consensus and details of monitoring are missing. EASL and Chinese Society of Hepatology (3, 5) recommend prophylaxis should continue for at least 18 months after stopping immunosuppression and monitoring should continue for at least 12 months after prophylaxis withdrawal. Only Chinese Society of Hepatology mentioned testing HBVDNA every 1-3 months after prophylaxis treatment. Other guidelines (1, 3, 4, 6, 7, 18) have no mention on frequency and tests advised for patients who finished monitoring as long as 12 months or 18 months after stopping immunosuppression treatment. However, there is no answer to the question on which indicators are the best, or the most sensitive to diagnosis of reactivation.

In this patient, ALT abnormality was the first manifestation, serum HBV DNA appeared 5 months later, and HBsAg turned positive 8 months later. It indicates that transaminase is an early, sensitive indicator for the detection of HBV reactivation. Therefore, transaminase testing should be performed to early diagnose of HBV reactivation in resource-limited areas or when HBsAg and HBV DNA cannot be detected promptly. However, in fact, when the patient visits the doctor with elevation of transaminases, we did not identify the cause as HBV reactivation immediately, considering that the cause might be viral hepatitis, drug-induced liver injury, nonalcoholic steatohepatitis (NASH), and autoimmune hepatitis, and after further examination the possibility of NASH was considered. After treatment for NASH (including oral diammonium glycyrrhizinate enteric-coated capsules and bicyclol tablets, adjusting dietary structure, appropriate activity, and weight loss), although the patient’s ALT decreased (possibly originating from bicyclol’s ALT lowering effect), AST didn’t turn to normal, which indicates persistent liver injury, until HBV becomes undetectable 3 months since TAF administration as showed in **Figure 1**. In short, the patient’s transaminases continued to be abnormal and did not return to normal until antiviral therapy took effect. From the characteristics of the response after treatment, we judged that the elevated transaminases at the time of the patient’s visit were caused by HBV. This means that ALT abnormalities may be the initial manifestation of HBV reactivation.

When considering HBV reactivation caused the transaminase elevation in this patient, there might be a puzzle: why serum HBV DNA remains undetectable (<20 IU/ml) while ALT is elevated? We suspect that the serum HBV DNA level was too low at that time, possibly below 20 IU/ml (lower limit of detection), so we could not detect it. In fact, when the HBV DNA test result is not ‘target not detected’, it means there may be HBV DNA at a low level. Candotti, D et al. (19) reported that even when donors were initially screened for highly sensitive HBsAg negative and highly sensitive HBV DNA < 3.4 IU/ml, HBV transmission still occurred by blood transfusion from 3 individuals with occult hepatitis B infection (OBI). In addition, even if this patient’s serum HBV DNA is indeed negative, there may still be HBV DNA replication in the liver tissue (20). Indeed, the detection of HBV DNA in liver tissue, instead of the serum HBV DNA, is the gold standard for the diagnosis of OBI in HBsAg negative individuals. Our guess could be further established if we performed liver biopsy at that time and detect HBV DNA in liver tissue. This reflects the diagnostic value of liver biopsy for HBV infection. In conclusion, we suggest that serum HBV DNA may not be a sensitive marker for the diagnosis of HBV reactivation and may lag behind changes in liver biochemistry.

Fortunately, this patient responded well to TAF treatment. After 4 weeks of antiviral treatment with TAF, virological response (HBV DNA <20 IU/ml) was obtained, and complete response was obtained after 12 weeks of treatment. The rapid response is due to the following factors: Firstly, because of timely surveillance, HBV reactivation was diagnosed early. Secondly, considering the safety, TAF was selected as anti-HBV medications. Thirdly, the patient’s lymphoma was in complete remission at the time of HBV reactivation, and she had no history of chronic liver diseases, which was the basis for achieving a complete response. Timely surveillance, diagnosis and intervention is the key to manage HBV reactivation. In terms of medication, current guidelines recommend entecavir (ETV), tenofovir disoproxil fumarate (TDF) and TAF as the first-line antiviral treatment (3,7.18). Seto W K et al. (8) reported that monitoring every 4 weeks after rituximab treatment in HBsAg-negative HBcAb-positive lymphoma patients is feasible and effective, and that HBVDNA became undetectable after 6 weeks (range, 4 to 12 weeks) of ETV treatment in all HBV reactivators. No hepatitis or liver failure related to HBV was observed during the study.

Studies (21, 22) showed elderly patients are risk factor for HBV reactivation. In our report the patient was 72-year-old, the competency of the adaptive immune and innate immunity function decreased. The weakened ability of immune response leading to disability of viral clearance might contribute to the HBV reactivation. HBV reactivation depends on host related factors, viral factors and factors related to immunosuppressive agent.

In summary, clinicians should be aware of HBV reactivation and late HBV reactivation for HBsAg - negative, HBcAb - positive patients who receiving B-cell monoclonal antibody agents. Eighteen months is not enough for the duration of monitoring. Regular monitoring with a frequency of 1-3 months is the basis for timely diagnosis and treatment of HBV reactivation. Liver biochemistry, HBV DNA and HBsAg can be preferred in monitoring indicators. Notably, ALT abnormalities may be the initial manifestation of HBV reactivation.

## Data availability statement

The original contributions presented in the study are included in the article/supplementary material. Further inquiries can be directed to the corresponding authors.

## Ethics statement

Written informed consent was obtained from the individual(s) for the publication of any potentially identifiable images or data included in this article.

## Author contributions

XG and TJ conducted writing, research, and study revision. SX was responsible for the Figure of the paper and contributed to the writing and study revision. JX and YY helped in revision and final approval of the draft manuscript. All authors contributed to the article and approved the submitted version.

## Acknowledgments

Thanks to Zhifeng Jiang for the continuous support.

## Conflict of interest

The authors declare that the research was conducted in the absence of any commercial or financial relationships that could be construed as a potential conflict of interest.

## Publisher’s note

All claims expressed in this article are solely those of the authors and do not necessarily represent those of their affiliated organizations, or those of the publisher, the editors and the reviewers. Any product that may be evaluated in this article, or claim that may be made by its manufacturer, is not guaranteed or endorsed by the publisher.
